# Analysing the Effect of Mutation on Protein Function and Discovering Potential Inhibitors of CDK4: Molecular Modelling and Dynamics Studies

**DOI:** 10.1371/journal.pone.0133969

**Published:** 2015-08-07

**Authors:** Nagasundaram N, Hailong Zhu, Jiming Liu, Karthick V, George Priya Doss C, Chiranjib Chakraborty, Luonan Chen

**Affiliations:** 1 Department of Computer Sciences, Hong Kong Baptist University, Kowloon Tong, Hong Kong; 2 Medical Biotechnology Division, School of Biosciences and Technology, VIT University, Vellore, Tamil Nadu, India; 3 Department of Bioinformatics, School of Computer and Information Sciences, Galgotias University, Greater Noida, Uttra Pradesh, India; 4 Key Laboratory of Systems Biology, Shanghai Institutes of Biological Sciences, Chinese Academy of Sciences, Shanghai, China; Wake Forest University, UNITED STATES

## Abstract

The cyclin-dependent kinase 4 (CDK4)-cyclin D1 complex plays a crucial role in the transition from the G1 phase to S phase of the cell cycle. Among the CDKs, *CDK4* is one of the genes most frequently affected by somatic genetic variations that are associated with various forms of cancer. Thus, because the abnormal function of the CDK4-cyclin D1 protein complex might play a vital role in causing cancer, CDK4 can be considered a genetically validated therapeutic target. In this study, we used a systematic, integrated computational approach to identify deleterious nsSNPs and predict their effects on protein-protein (CDK4-cyclin D1) and protein-ligand (CDK4-flavopiridol) interactions. This analysis resulted in the identification of possible inhibitors of mutant CDK4 proteins that bind the conformations induced by deleterious nsSNPs. Using computational prediction methods, we identified five nsSNPs as highly deleterious: R24C, Y180H, A205T, R210P, and R246C. From molecular docking and molecular dynamic studies, we observed that these deleterious nsSNPs affected CDK4-cyclin D1 and CDK4-flavopiridol interactions. Furthermore, in a virtual screening approach, the drug 5_7_DIHYDROXY_ 2_ (3_4_5_TRI HYDROXYPHENYL) _4H_CHROMEN_ 4_ONE displayed good binding affinity for proteins with the mutations R24C or R246C, the drug diosmin displayed good binding affinity for the protein with the mutation Y180H, and the drug rutin displayed good binding affinity for proteins with the mutations A205T and R210P. Overall, this computational investigation of the *CDK4* gene highlights the link between genetic variation and biological phenomena in human cancer and aids in the discovery of molecularly targeted therapies for personalized treatment.

## Introduction

Cyclin-dependent kinases (CDKs) drive cell cycle progression, control transcriptional regulation processes and maintain cell proliferation. Irregular entry into the cell cycle and uncontrolled cell proliferation are hallmarks of cancer [1]. Hence, it is not surprising that the dysregulation of CDKs might play a vital role in tumorigenesis. The CDK4-cyclin D1-p16 retinoblastoma protein (RB1) pathway (CDK4 pathway) promotes the G1-S cell cycle transition and is commonly dysregulated in most cancers. The CDK4-cyclin D1 complex acts as an essential regulator in the G1-S phase transition of the cell cycle process. The CDKs and cyclins that are most frequently affected by somatic nucleotide alterations in various cancers are CDK4 and cyclinD1. Thus, abnormality of the CDK4/cyclin D1 pathway plays a major role in oncogenesis; hence, CDK4 can be genetically tested as a valid molecular therapeutic target. In 1996, Bradley et al. observed the inhibition of CDK4 by the flavonoid compound flavopiridol in breast cancer cell lines [2]. Flavopiridol was the first drug identified as a potent tumour suppressor in several lung and breast cancer cell lines [3]. Various studies have highlighted that flavopiridol has the capability to prevent the proliferation of a broad range of cell lines, leukaemias, lymphomas and human tumours [4, 5]. Several clinical trials have been completed up to the phase II level with various regimens. To date, several inhibitors with varying selectivity for members of the CDK family have been identified. CDK4 inhibitors are considered the most attractive therapeutic targets because of their ability to control tumour growth with minimal toxicity.

In more than 90% of melanoma cases, genomic variations associated with CDK4 pathway activation are present, as determined in human and mouse models of melanoma [6, 7]. A common type of genetic variation in the human genome is the single nucleotide polymorphism (SNP) [8]. SNPs are the naturally occurring nucleotide variability in the human genome and play a significant role in the phenotypic variability that differentiates individuals within a given species. SNPs can occur in both coding and noncoding regions of the genome and generate polymorphic variation in expressed amino acid sequences that affects protein structure and function. In the coding region of the genome, SNPs are mainly classified into two types: synonymous and non-synonymous (nsSNPs). Nonsynonymous SNPs can change the physicochemical properties of a protein residue, thereby disturbing protein stability and dynamics, affecting normal interactions with other molecules, and hindering stable complex formation with binding partners [9–12].

Protein-protein interacting interfaces are usually referred to as binding hot spots of proteins. These regions are charged, structurally conserved and highly polar and are surrounded by hydrophobic residues, which are the residues that are mostly involved in the binding [13]. Deleterious genetic variation may affect the electrostatic nature of protein surfaces and introduce harmful effects, such as changes in stability or folding, altering binding partner specificity and affinity and changing protein function [13]. The identification of harmful nsSNPs helps uncover plausible molecular mechanisms underlying disease phenotypes and helps in the development of suitable inhibitors to target the mutant proteins. Individual nsSNPs are causation for significant changes in drug disposition and efficacy. Because nsSNPs can occur in drug binding proteins, they can affect treatment response or produce adverse effects.

In this study, we employed efficient computational prediction methods to identify deleterious nsSNPs in the *CDK4* gene. We then evaluated the effects of deleterious CDK4 variants on CDK4-Cyclin D1 protein interactions and drug binding. Protein structure-based virtual screening analysis was performed to identify suitable inhibitors of mutant CDK4 proteins. Furthermore, atomic level studies were performed by molecular dynamics (MD) simulations to better understand the effects of deleterious variants on CDK4-Cyclin D1 complex formation and to check the binding efficacies of selected inhibitors for the mutant proteins.

## Material and Methods

### Datasets

The nsSNPs in the *CDK4* gene were extracted from the dbSNP [14] and UniProt [15] databases. The amino acid length of CDK4 and cyclinD1 protein sequence is 306 and 271 residues respectively. For structure analysis, the CDK4-Cyclin D1 protein complex crystal structure was obtained from the PDB database (PDB ID: 2W96) [16]. For virtual screening, flavopiridol-like compounds were retrieved from the DrugBank database [17].

### Deleterious nsSNP prediction tools

Different computational methods have been previously developed for the prediction of phenotypic effects of nsSNPs. In this study, three variation tolerance prediction methods, SIFT [18], PolyPhen 2 [19], and I-Mutant 3.0 [20], were used following the same protocol, in which nsSNPs are first labelled with amino acid properties according to the changes they may have on protein structure or function. The pathogenicity of the nsSNPs is decided based on the resultant vectors calculated using the individual tools. The prediction by each method is generally based on evolutionary information and a combination of protein structural and/or functional parameters and multiple sequence alignment-derived information. SIFT calculates a tolerance index score for a particular residue substitution. This algorithm first generates multiple sequence alignments with a large set of homologous amino acid sequences and predicts a tolerance index for each residue, ranging from zero to one. PolyPhen 2 predicts the possible effects of amino acid substitutions on protein structure and function using straightforward physical and evolutionary comparative considerations. PolyPhen 2 searches for multiple sequence alignments of homologous protein sequences, 3D protein structures and residue contact information from secondary structure databases. Based on this information, PolyPhen 2 calculates PSIC scores for each of the two variants and computes the differences between the PSIC scores. If the predicted score is higher for a particular substituted amino acid, that substitution is likely to have a higher functional effect on protein structure/function. The machine learning method I-Mutant 3.0 utilises support vector machines (SVMs) for classification. It is built on unsupervised classification using support vector machines and trained on the most comprehensive dataset derived from ProTherm for the prediction of protein stability changes resulting from nsSNPs. The energy difference between the native and the variant protein was calculated based on the Gibbs free energy value and the predicted free energy change is denoted by a DDG value.

### Docking and virtual screening

AutoDock is one of the most widely accepted docking software programs available and requires a set of preparation steps for general screening [21]. Included in this process are the preparations of acceptable ligands and a receptor macromolecule, calculation of maps and creation of folders for each ligand.AutoDockVina1.1.2 is a new program for molecular docking and virtual screening and approximately two orders of magnitude faster than AutoDock4 [22]. AutoDockVina 1.1.2 was used for all dockings in this study. In general, the docking parameters for AutoDockVina were kept to their default values. In vina, the size of the docking grid was 63 Å×47 Å×40 Å, which encompassed all the native and mutant protein structure.For high throughput virtual screening, the VcPpt tool was used. VcPpt is an independently developed extension for AutoDockVina and is a software package for flexible protein-ligand docking built by the Biochem Lab solution. This package can perform high-throughput docking for a given compound with Vina, and the output is PDB files of ligands ranked based on their binding energies and positions.

### Molecular dynamic simulations

Molecular dynamics simulations of native and mutant protein-protein and protein-ligand complexes were performed using Gromacs 5.0 software [23]. The force field used for the simulation was Gromos96 43a1 [24, 25]. The structures were solvated using a simple point charge (SPC) water molecules in a box with a dimension of 52.0 Å size. The number of water molecules added into the boxes differed and it depends on the concerned system. For protein-protein complexes43918, 43931, 43926, 43930, 43926 and 43932 water molecules were added to respective native, R24C, Y180H, A205T, R210P and R246C simulation boxes and for protein-ligand complexes43915, 43919, 43888, 43893, 51929 and 43918 water molecules were added to respective native, R24C, Y180H, A205T, R210P and R246C simulation boxes. At physiological pH, protein-protein and protein-ligand complexes were negatively charged, and counter ions (Na+) were added to make the simulation system neutral. Then, the system energy was minimised by utilising the steepest descent method. After minimisation, three different steps were employed in the MD simulation: namely, heating, equilibration, and production. An NPT ensemble (constant number of particles, pressure, and temperature) was performed for 50000 ps at 300 K followed by an NVT ensemble (constant number of particles, volume and temperature) that was performed at 300 K [26]. Then, the production of molecular dynamics simulation trajectories was performed at 300 K for 50 ns. The Linear Constraint Solver (LINCS) algorithm was used to constrain the covalent bonds [27]. The Particle Mesh Ewald (PME) method was used to calculate electrostatic interactions [28]. The cutoff radii for van der Waals and Coulomb interactions were fixed at 14.0 and 10.0 Å, respectively.

The trajectory potentials obtained from each MD simulation were thoroughly investigated by using GROMACS utilities [29]. The utilities g_rms, g_rmsf, g_hbond, g_mindist and g_sas were used to plot graphs. The g_rms program calculates the root mean square deviation (RMSD) for specified atoms in a protein molecule with respect to a reference structure by fitting the structure to least square level with the reference structure. The g_rmsf program computes the root mean square fluctuation (RMSF) (i.e. standard deviation) of C-alpha atomic position of a protein molecule after fitting to a reference structural frame. The g_hbond program calculates number of hydrogen bonds formed between two molecules based on simple geometric criteria. This program analyzes the possibilities for hydrogen bond formation between all possible acceptors (A) and donors (D). The most accepted geometrical distance for a hydrogen bond formation between molecules is <2.5 Å and between hydrogen and the acceptor and a donor-hydrogen-acceptor angle of between 90° and 180°. The program g_mindist calculates the minimum distance between the atoms of two different molecules during simulation time. It also calculates the number of contacts made between two molecules within a certain radius r_max_. The g_sas computes total solvent accessible surface area, hydrophilic and hydrophobic interaction of protein molecules. To check whether the systems follow constant NVT or NPT ensembles, differences in total, potential, and kinetic energies, pressure and temperature were calculated as a function of simulation time throughout the simulation period. The numbers of H-bond formation and the minimum distance between protein-protein and protein-ligand complexes were calculated to explain the stability of the complexes. SASA analysis was performed to identify the solvent traceable area of a molecule, and all graphs were generated using the XM grace tool [30].

### Principle compound analysis

Essential Dynamics (ED) method was used to calculate the eigenvectors and eigenvalues, and their projection along the first two principal components [31]. ED or the principle component analysis (PCA) is a method that reduces the complexity of the data and extracts the different modes involved in the motion of the protein molecule [31]. In the process of ED calculation, a covariance matrix was generated from the trajectories after the elimination of translational and rotational movements. By diagonalizing the covariance matrix, a set of eigenvectors and eigenvalues were generated. For each eigenvector, one corresponding eigenvalue produced explains the energetic contribution of each component to the motion. The protein molecular segments that are responsible for the most significant collective motions can be acknowledged through PCA. Backbone C-alpha bonds trajectories were obtained and analyzed by using g_covar and g_anaeig of GROMACS inbuilt tool.

## Results

### Prediction of deleterious nsSNPs using the SIFT, Polyphen2 and I-Mutant 3.0 programs

**Fig 1:**
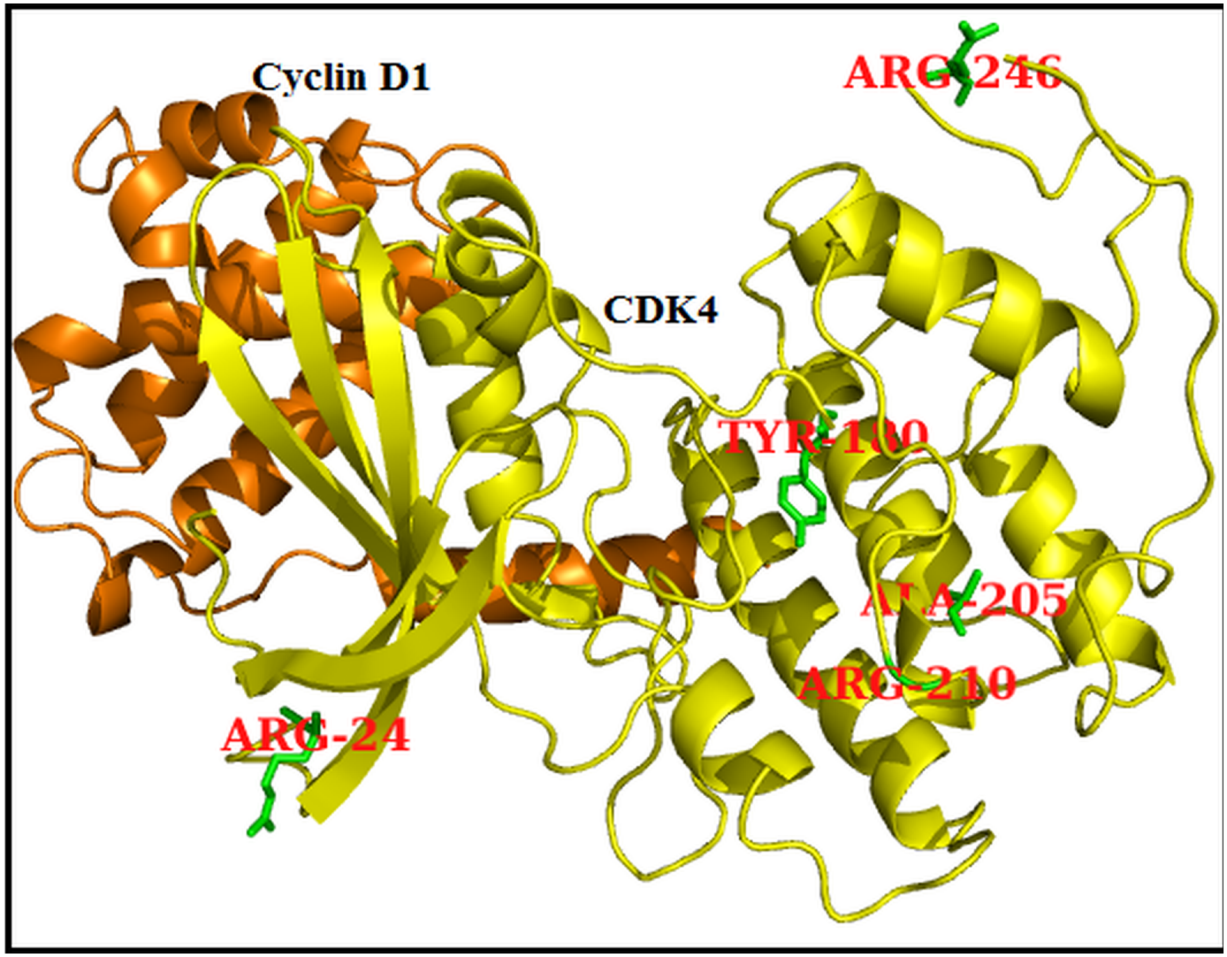
CDK4 (Yellow) in complex with CyclinD1 (Orange) protein and all the five highly deleterious variants are highlighted in stick model (Green colour).

Out of 20 nsSNPs submitted to SIFT, 11 nsSNPs (corresponding to R24C, R24H, Y180H, A205T, R209C, R210P, R246C, P251H, R255H, V260E, and H296Y) were predicted to be deleterious, with SIFT scores <0.05 (Table 1). Of these, sixnsSNPs (R24C, R24H, Y180H, A205T, R209C, and R210P) were considered to be highly deleterious, with SIFT scores of 0.00 (Table 1). PolyPhen 2 predicted ninensSNPs that would deleteriously affect protein structure and function, and the remaining 12 nsSNPswere characterised as benign. Of these nine deleterious nsSNPs, one nsSNP (Y180H) was predicted to be highly deleterious, with a SIFT score of 1.000 (Table 1). SIFT and PolyPhen 2 has been shown to perform well in predicting deleterious nsSNPs compared to other computational tools [32]. To improve the accuracy of deleterious nsSNP prediction, along with SIFT and PolyPhen 2, we used I-Mutant 3 to predict the deleteriousness of nsSNPs that affect protein stability. All of the nsSNPs analysed in SIFT and PolyPhen 2.0 were also submitted to the I-Mutant 3.0 server. I-Mutant 3.0 identified 15 nsSNPs as deleterious. By comparing the predictions by all three tools, we identified five nsSNPs (R24C, Y180H, A205T, R210P, and R246C) to be highly deleterious (Fig 1) and subjected these to further structural and functional analysis.

**Table 1:** List of nsSNPs present in *CDK4* gene and their predicted SIFT, PolyPhen2 and I-Mutant3 scores.

SNP ID	Amino acid variation	SIFT	PolyPhen2	I-Mutant3
**rs11547328**	**R24C**	**0.00**	**0.737**	**Disease**
rs104894340	R24H	**0.00**	0.324	**Disease**
rs199609381	P26H	0.15	0.015	**Disease**
rs376139539	P40H	0.05	0.174	**Disease**
rs144890720	N41S	0.15	0.002	**Disease**
rs200213586	A65V	0.25	**0.603**	**Disease**
rs3211612	R82Q	0.09	**0.990**	**Disease**
rs34386532	R122H	0.55	0.001	**Disease**
rs150281463	V137L	0.18	0.001	**Disease**
rs151103937	V176I	0.06	**0.956**	Neutral
**rs375372343**	**Y180H**	**0.00**	**1.000**	**Disease**
**rs368013594**	**A205T**	**0.00**	**0.987**	**Disease**
rs140644696	R209C	**0.00**	0.258	**Disease**
**rs373619077**	**R210P**	**0.00**	**0.995**	**Disease**
**rs370258992**	**R246C**	**0.01**	**0.995**	**Disease**
rs372604524	R246H	0.09	0.016	Neutral
rs143670820	P251H	**0.01**	**0.755**	Neutral
rs144657355	R255H	**0.01**	0.004	Neutral
rs201617914	S259L	0.18	0.004	Neutral
rs200215596	V260E	**0.01**	0.356	Neutral
rs2227954	H296Y	**0.03**	0.155	**Disease**

Highly deleterious by SIFT, Probably and possibly damaging by PolyPhen2 and disease/neutral by I-Mutant3 prediction were highlighted in bold

### CDK4-Flavopiridol binding analysis in the occurrence of deleterious nsSNPs in CDK4 protein

Flavopiridol has been suggested to inhibit the *in vitro* kinase activity of the CDK4 protein [2]. Amino acid variation may affect the inhibitory action of flavopiridol on the CDK4 protein. Numbers of studies have been performed to find an ATP-competitive inhibitor that binds specifically to the CDK4 protein [33–37]. Additionally, further studies have attempted to optimise inhibitor binding and specificity for CDK4 using structure-based design methods [38–40]. In this study, we examined the binding affinity of flavopiridol with native and mutant CDK4 proteins using AutodockVina, a computational docking program. Before performing the docking analysis, ATP binding sites of the native CDK4 protein were identified. The amino acid residues present in the ATP binding clefts of CDK4 are ILE12, VAL20, ALA33, VAL77, PHE93, GLU94, HIS95, VAL96, GLN98, ASP99, THR102, GLU144, LEU147, ALA157 and ASP158.Computational docking analysis also indicated the inhibitory action of flavopiridol with CDK4, as observed in *in vitro* studies.e., the inhibitor flavopiridol binds exactly at the ATP binding site of the native CDK4 protein (S1 Fig). However, flavopiridol binds residues outside of the ATP-binding cleft in mutant CDK4 structures (S1 Fig). A change in the binding residues will indeed affect the complementarities between the mutant proteins and flavopiridol. Non-covalent interactions and shape complementarity are important factors for the maintenance of protein-ligand affinity. Non-covalent bonds, such as van der Waals contacts, electrostatic forces and hydrogen bonds, are the primary forces involved in protein-ligand interactions. Calculating the interaction energies of non-covalent bonds is vital to understanding the binding ability of the ligand molecule. The number of hydrogen bonds arising between the protein and ligand was computed using AutodockVina. The binding energies between the CDK4 (native and mutant) proteins and the inhibitor molecule flavopiridol were calculated (Table 2) to be -8.8 kcal/mol, -7.7 kcal/mol, -7.1 kcal/mol, -7.3 kcal/mol, -7.4 kcal/mol and -7.1 kcal/mol for the native, R24C, Y180H, A205T, R210P and R246C complexes, respectively. The binding energy of the native complex displayed the best interaction and complete inhibition by the flavopiridol compound. This docking analysis gives a “theoretical quantitative” assessment of the binding efficiencies of CDK4 native and mutant proteins with the cancer drug flavopiridol.

**Table 2:** Binding energies of CDK4 native and mutant proteins with drug flavopiridol.

Native & Mutant proteins	Binding Energy(Kcal/mol)	Number of H-Bonds	Residues forming H-Bonds with Ligand	Interacting Residues
**Native**	-8.8	3	VAL14,LYS142	ILE12, GLY13,VAL14, GLY15, VAL20,LYS35, ASP99, LYS142
**R24C**	-7.7	3	VAL14,LYS142	ILE12, GLY13,VAL14, GLY15, VAL20,LYS35, ASP99, LYS142
**Y180H**	-7.1	1	ARG5	ARG5,GLU92,LEU143,LYS147
**A205T**	-7.3	3	VAL137,ARG163	GLU56,GLU59,ARG163,TYR191,VAL137
**R210P**	-7.4	3	VAL137, ARG163	GLU56, LEU59,VAL137, ARG163,TRY191
**R246C**	-7.1	3	VAL137, ARG163	LEU23,ARG26,HIS68,ARG126, PHE130,ALA133

### CDK4 mutant protein structures based virtual screening and docking studies

**Fig 2:**
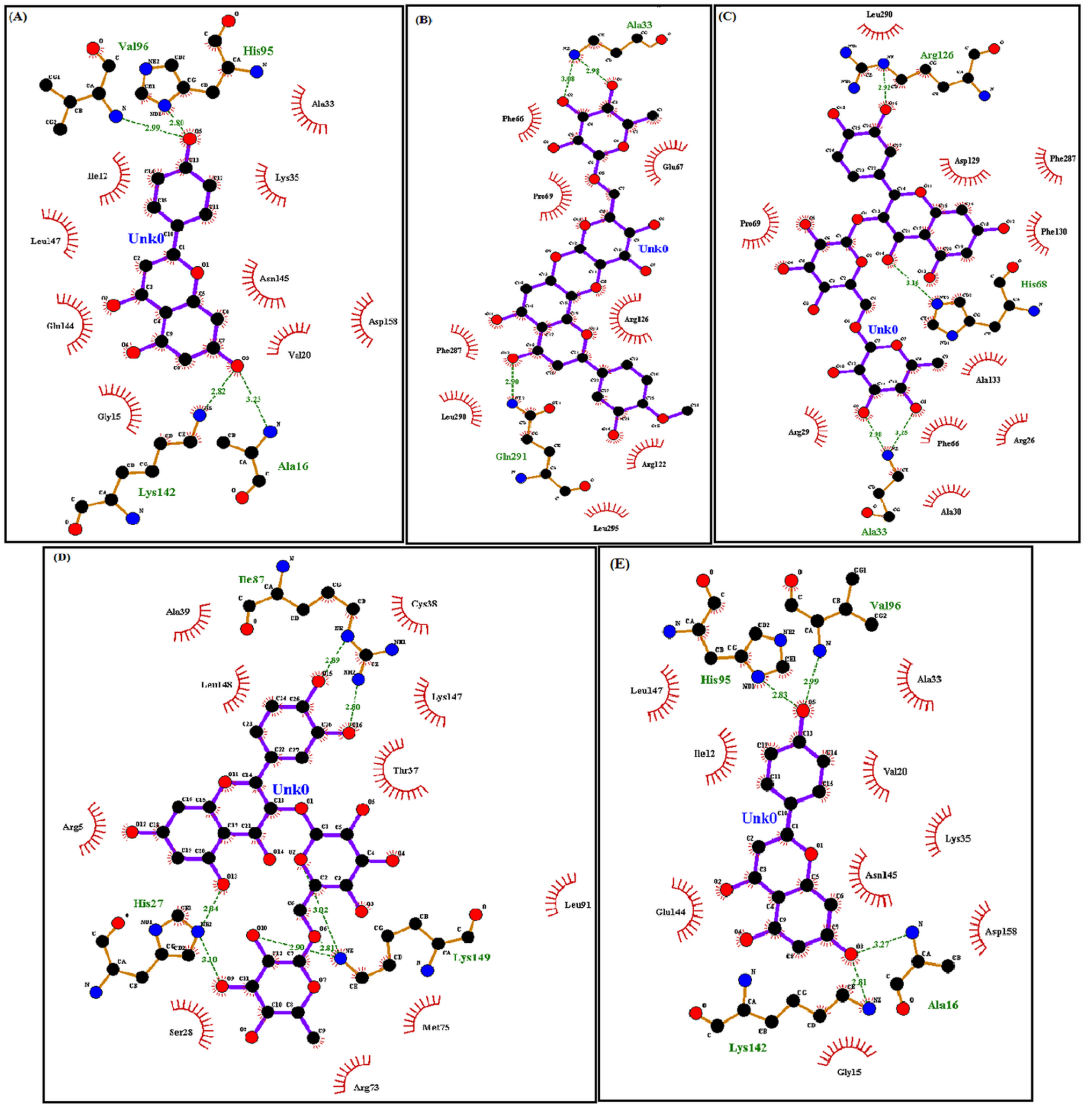
Ligplot analysis of CDK4 mutant proteins with virtual compounds. (A) Ligplot showing interaction between mutant type R24C and 5_7_DIHYDROXY_2_ (3_4_5_TRIHYDROXYPHENYL) _4H_CHROMEN_4_ONE. (B) Ligplot showing interaction between mutant model Y180H and Diosmin. (C) Ligplot showing interaction between mutant type A205T and Rutin. (D) Ligplot showing interaction between mutant type R210P and Rutin. (E) Ligplot showing interaction between mutant type R246C and 5_7_DIHYDROXY_2_ (3_4_5_TRIHYDROXYPHENYL) _4H_CHROMEN_4_ONE.

Nonsynonymous SNPs play a vital role in the diverse responses to therapeutic treatment in human populations, influencing efficacy and toxicity by affecting the drug-binding pocket of target proteins. Virtual screening is the fastest and most accurate method for identifying novel drug-like compounds on the basis of target structures [41, 42].It has an advantage over any *de novo* design method because retrieved hits can be easily obtained for biological testing. Docking is a computational method used to predict binding affinities between a target protein and a ligand. Docking follows a search pattern to identify appropriate confirmations and a score that measures the affinity of various conformations [43, 44]. For virtual screening, we retrieved 19 similar compounds, such as flavopiridol, from the DrugBank database (Table 3). Subsequently, docking analysis was performed between mutant CDK4 proteins (R24C, Y180H, A205T, R210P, and R246C) and the screened compounds (Table 4). Among the 19 compounds docked, R24C and R246C mutant proteins displayed good binding to the drug 5_7_DIHYDROXY_2_ (3_4_5_TRIHYDROXYPHENYL) _4H_CHROMEN_4_ONE,with a binding energy of -8.3 kcal/mol and -8.2 kcal/mol, forming four hydrogen bonds with R24C and R246C mutant proteins, respectively.This compound interacts with the ATP binding residues of both R24C (ILE12, VAL20, HIS95, VAL96, GLU144, and LEU147) and R246C (ILE12, HIS95, VAL96, GLU144 and LEU147) mutant protein structures (Fig 2A and 2E).Diosmin displayed a good affinity for the mutant protein structure Y180H and obtained, with a high binding energy of -7.7 kcal/mol. Diosmin formed three hydrogen bonds with Y180H and interacted with the ATP binding residue ALA33 (Fig 2B). Rutin displayed good binding with the mutant structures A205T and R210P and obtained the highest binding energy of -8.6 and -8.3 kcal/mol, respectively. Rutin formed four hydrogen bonds with A205T and six hydrogen bonds with R210P and interacted with the ATP binding residue ALA33 of the A205T mutant protein. Notably, no interaction was observed with the ATP binding residues of R210P (Fig 2C and 2D).

**Table 3:** List of virtually selected compounds similar to flavopiridol from DrugBank database.

Accession number	Name	Chemical formula	Class	Groups	Original uses
**DB07024**	2_(3_4_DIHYDROXYPHENYL)_8_(1_1_DIOXIDOISOTHIAZOLIDIN_2_YL)_3_HYDROXY_6_METHYL_4H_CHROMEN_4_ONE	C_19_H_17_NO_7_S	Flavonoids	Experimental	-
**DB07453**	2_PHENYL_4H_BENZO[H]CHROMEN_4_ONE	C_19_H_12_O_2_	Flavonoids	Experimental	-
**DB07776**	2_PHENYL_4H_CHROMEN_4_ONE	C_15_H_10_O_2_	Flavonoids	Experimental	-
**DB08230**	3_7_3'_4'_TETRAHYDROXYFLAVONE	C_15_H_10_O_7_	Flavonoids	Experimental	-
**DB08230**	5_7_DIHYDROXY_2_(3_4_5_TRIHYDROXYPHENYL)_4H_CHROMEN_4_ONE	C_15_H_10_O_7_	Flavonoids	Experimental	-
**DB06927**	5_HYDROXY_2_(4_HYDROXYPHENYL)_1_BENZOFURAN_7_YL]ACETONITRILE	C_16_H_11_NO_3_	Benzofurans	Experimental	-
**DB02205**	6_(1_1_Dimethylallyl)_2_(1_Hydroxy_1_Methylethyl)_2_3_Dihydro_7h_Furo[3_2_G]Chromen_7_One	C_19_H_22_O_4_	Coumadin’s and Derivatives	Experimental	-
**DB01838**	6_4'_Dihydroxy_3_Methyl_3'_5'_Dibromoflavone	C_16_H_10_Br_2_O_4_	Flavonoids	Experimental	-
**DB02593**	7_8_Dihydroxy_1_Methoxy_3_Methyl_10_Oxo_4_10_Dihydro_1h_3h_Pyrano[4_3_B]Chromene_9_Carboxylic Acid	C_15_H_14_O_8_	Benzopyrans	Experimental	-
**DB06732**	Beta_Naphthoflavone	C_19_H_12_O_2_	Flavonoids	Experimental	Putative chemo preventive agent
**DB06726**	Bufuralol	C_16_H_23_NO_2_	Benzofurans	Experimental, investigational	Non-selective adrenoceptor blocking agent
**DB04886**	Calanolide A	C_22_H_26_O_5_	Coumarins and Derivatives	Investigational	Non-nucleoside reverse transcriptase inhibitor (NNRTI)
**DB08995**	Diosmin	C_28_H_32_O_15_	Not Available	approved	Oral phlebotropic drug used to venous disease
**DB01547**	Drotebanol	C_19_H_27_NO_4_	Morphinans	experimental, illicit	-
**DB01148**	Flavoxate	C_24_H_25_NO_4_	Flavonoids	Approved	Used in various urinary syndromes and as an antispasmodic
**DB01852**	Kaempherol	C_15_H_10_O_6_	Flavonoids	Experimental	-
**DB02375**	Myricetin	C_15_H_10_O_8_	Flavonoids	Experimental	-
**DB04216**	Quercetin	C_15_H_10_O_7_	Flavonoids	Experimental	Used as an antioxidant
**DB01698**	Rutin	C_27_H_30_O_16_	Flavonoids	Experimental	Used to decrease capillary fragility

**Table 4:** Virtual compounds with highest binding energy with CDK4 mutant proteins.

CDK4 Mutant Proteins	Binding Drug	Binding Energy (Kcal/mol)	Number of H-Bonds	Residues Forming H-Bonds with Ligand	Interacting Residues
**R24C**	5_7_DIHYDROXY_2_(3_4_5_TRIHYDROXYPHENYL)_4H_CHROMEN_4_ONE	-8.3	4	ALA16,HIS95,VAL96,LYS142	ILE12, GLY15,ALA16, VAL20, LYS35,HIS95,VAL96,LYS142, GLU144,ASN145, LEU147
**Y180H**	Diosmin	-7.7	3	ALA33, GLN291	ALA33,PRO69,ARG122,ARG126, GLN291
**A205T**	Rutin	-8.6	4	ALA33,HIS68,ARG126	ARG26,ALA33,PHE66, HIS68,ARG126
**R210P**	Rutin	-8.3	6	HIS27,ILE87, LYS149	ARG5,HIS27,ILE87, ARG73, LEU94,LYS149
**R246C**	5_7_DIHYDROXY_2_(3_4_5_TRIHYDROXYPHENYL)_4H_CHROMEN_4_ONE	-8.2	4	ALA16,HIS95,VAL96,LYS142	ILE12,GLY15,ALA16,LYS35,HIS95,VAL96,LYS142,GLU144,ASN145, LEU147

### Molecular dynamics simulation studies of the CDK4-Cyclin D1 complex

To examine the structural and functional consequences of deleterious nsSNPs in the CDK4-Cyclin D1 complex, we analyzed the 50ns molecular dynamics simulation trajectories of native (CDK4-Cyclin D1) and mutant (R24C, Y180H, A205T, R210P and R246C) complexes.

#### Equilibration and Stability checking of native and mutant CDK4-Cyclin D1 complexes

The root mean square deviations (RMSDs) of protein backbone atoms of native and mutant CDK4-Cyclin D1 complexes were analysed. For all of the simulations, the energy minimised protein complexes were taken as a starting reference. To obtain all-atom levels in detail, MD simulations were repeated twice for native and mutant complexes for a time period of 50 ns. No significant changes were observed from the repeated MD simulation trajectories of native and mutant CDK4-Cyclin D1 complex structures. The simulated CDK4-Cyclin D1 complex backbone atoms were aligned with RMSDs of less than 3.5 A°.

After 35000 ps, all of the native and mutant protein complexes attained an equilibrated state. Native complexes obtained an RMSD ~0.3 nm in the equilibrated state, but the mutant complex Y180H displayed a different pattern with an RMSD value of ~0.5 to ~0.6 nm until the end of the simulation period. Mutant complexes R24C, A205T, R210P and R246C obtained an RMSD of~0.4 to ~0.5 nm in the equilibrated state (S2 Fig). Although minor deviations were observed during simulation periods, the stable trajectory formations in the equilibrium state provide a suitable basis for further analysis.

#### Calculation of amino acid fluctuations in native and mutant CDK4 proteins

The C-alpha root means square fluctuation (RMSF) values for each amino acid of CDK4 proteins were calculated. Graphs were plotted, for native and mutant proteins, from the values from the last 10ns of trajectories to obtain the amino acid mobility of each protein (S3 Fig). We found that all of the mutant proteins, R24C, Y180H, A205T, R210P, and R246C, had different fluctuation patterns from the native CDK4 protein. Mutation induced changes in the flexibility of the proteins, indicating that mutations may affect the function of the proteins by altering CDK4-Cyclin-D1 interactions.

#### Hydrogen bonding and minimum distance between CDK4 and Cyclin D1 in native and mutant complexes

**Fig 3:**
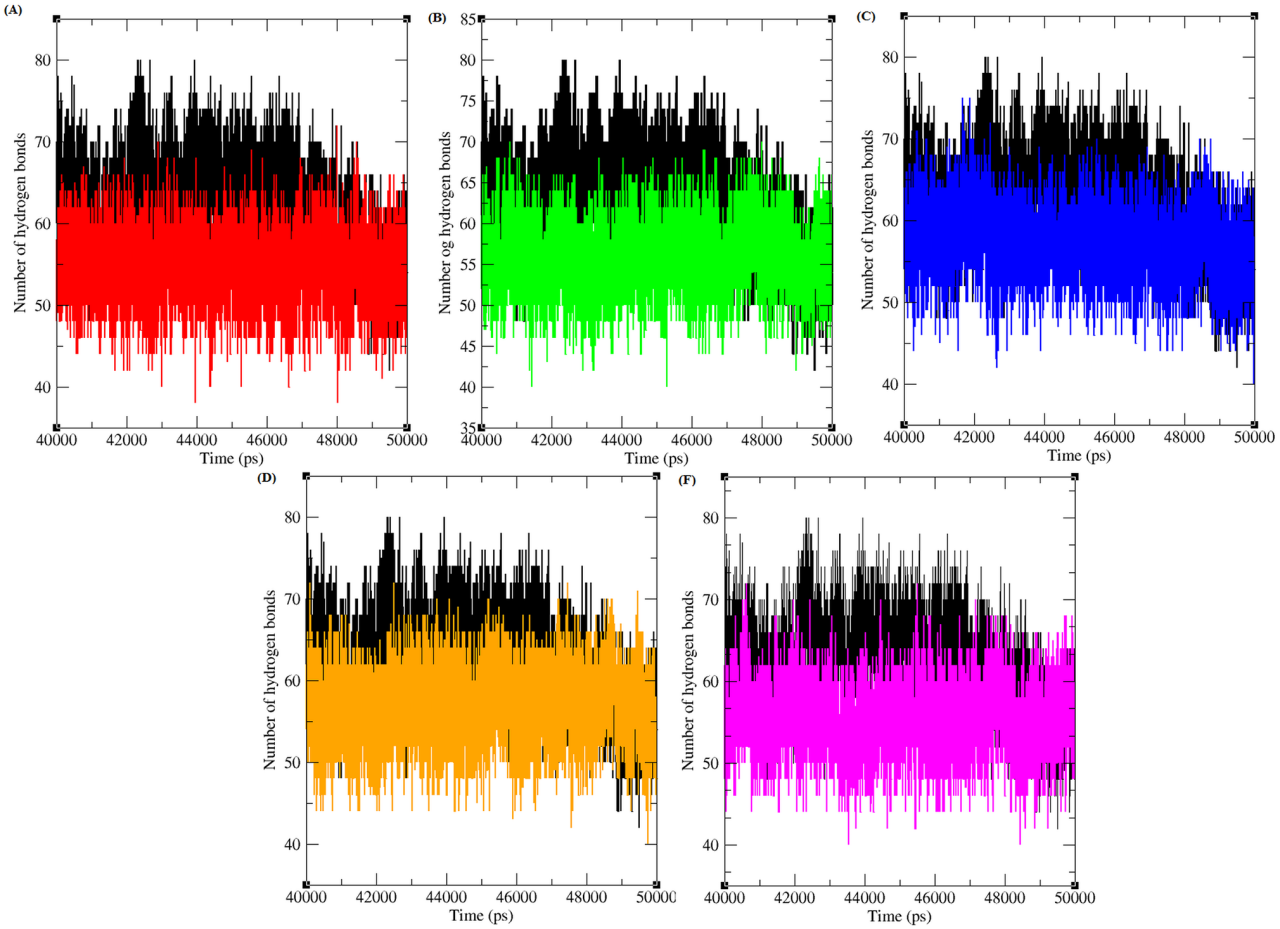
Total number of hydrogen bond formed between CDK4 and cyclin D1 protein in native and mutant state. Black, Red, Green, Blue, Orange and Pink lines indicate native, R24C, Y180H, A205T, R210P and R246C protein complexes respectively.

Hydrogen bonds arise when a hydrogen atom covalently bound to a molecule interacts with an electronegative atom within the same molecule or in a different molecule. As they are responsible for maintaining the stability of protein structure, determining hydrogen bonds significantly reveals the stability of a protein or the stability between the proteins [45]. Therefore, assessing the number of hydrogen bonds between protein-protein interacting regions is essential to gain knowledge regarding the stability of the molecules. Fig 3 depicts the number of hydrogen bonds formed between CDK4-Cyclin D1 proteins in native and mutant states. Native complexes of CDK4-Cyclin D1protein exhibit an average of ~ 45 to ~80 hydrogen bonds throughout the last 10ns simulation period. The maximum number of hydrogen bonds formed between CDK4-Cyclin D1 in the mutant complexes R24C, Y180H, A205T, R210P and R246C were ~ 40 to ~65, ~ 40 to ~70, ~ 45 to ~75, ~ 40 to ~70, and ~ 40 to ~70, respectively, in the last 10 ns of the simulation period. The mutant protein complexes R24C, Y180H, A205T, R210P, and R246C had fewer hydrogen bonds than did the native protein complex. The decrease in the number of hydrogen bonds formed between mutant CDK4-Cyclin D1complexes indicates the deleterious effects of amino acid substitution and their ability to reduce the number of hydrogen bonds formed between CDK4 and Cyclin D1 proteins.

**Fig 4:**
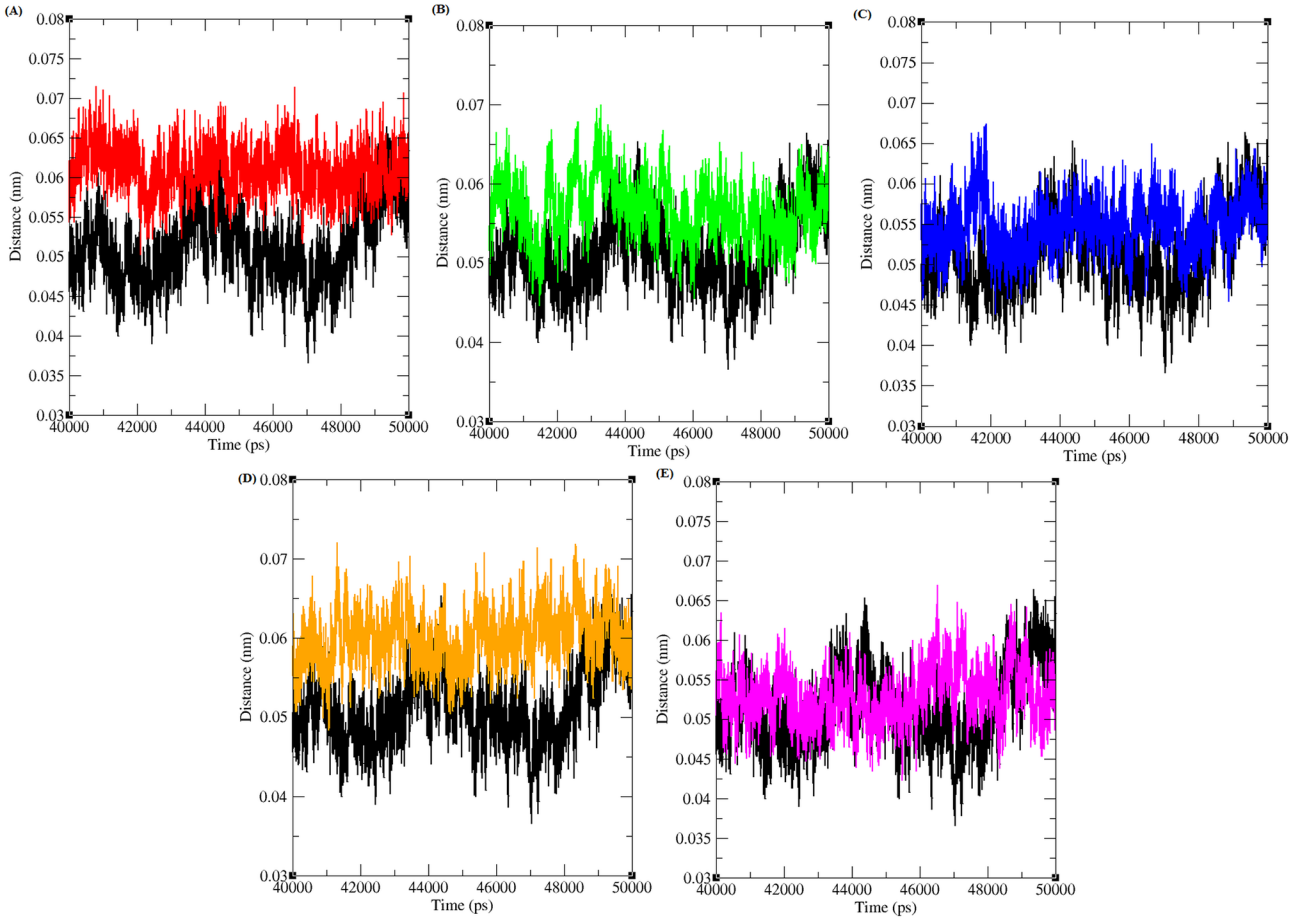
Minimum distance between CDK4 and cyclin D1 protein in native and mutant state. Black, Red, Green, Blue, Orange and Pink lines indicate native, R24C, Y180H, A205T, R210P and R246C protein complexes respectively.

Furthermore, the minimum distance between CDK4 and the Cyclin D1 protein was computed for both the native and mutant complexes (Fig 4). The minimum distance between native CDK4 and Cyclin D1 was observed to be ~ 0.035 to ~0.065 nm in the last 10 ns of the simulation period. However, for the mutant complexes R24C, Y180H, A205T, R210P and R246C, increases in the distance were observed to be ~ 0.05 to ~0.07 nm, ~ 0.045 to ~0.07, ~ 0.045 to ~0.067, ~ 0.05 to ~ 0.07 and ~ 0.045 to ~0.067, respectively. From the minimum distance analysis, we infer that the distance between mutant CDK4-Cyclin D1 proteins is greater than in the native complex.

#### Effects of nsSNPs in the SASA of native and mutant CDK4 proteins

The protein surface that is traced by a solvent molecule is referred to as the solvent-accessible surface area (SASA). The solvation effect plays a significant role in maintaining protein stability and folding. Likewise, the solvation effect accompanies protein-protein interaction processes and protein structure modifications. This solvation effect can be measured using explicit solvent models; MD simulations use a sphere of water molecules [46]. SASA was calculated for both the native and mutant CDK4 proteins. From S4 Fig, it was observed that the native CDK4 protein had an SASA of ~18.5 nm^2^ to ~ 21.5 nm^2^ in the last 10ns simulation period but that the mutant structures R24C, Y180H, A205T, R210P, and R246Chad different SASAs, of ~19 nm^2^to ~21.5 nm^2^, ~19 nm^2^ to ~21.5 nm^2,^ ~19 nm^2^ to ~22 nm^2,^ ~18.5 nm^2^ to ~22 nm^2^and ~18.5 nm^2^ to ~21.5 nm^2^, respectively. Compared with the native protein, all five mutant proteins had different SASAs. The differences in the SASAs of the mutant proteins indicated that there might be a shift in the amino acid residues from the buried region to the accessible area or vice versa, and this may be the reason for the reduced affinity between the mutant CDK4 and Cyclin D1 proteins.

#### Principal component analysis

PCA was performed on all the six trajectories of CDK4-Cyclin D1 native and mutant complexes to examine the overall motion of the protein molecules. Diagonal covariance matrices were built over the C-alpha atoms of the protein for each trajectory. The eigenvalues obtained through the diagonalization of the covariance matrix elucidates the atomic contribution on the motion. Similarly, the eigenvectors explain a collective motion accomplished by the particles. The spectrum of the corresponding eigenvalues indicated the level of fluctuation and dynamic behaviour of protein molecule in the system and confined within the first two eigenvectors. The trace values for native, R24C, Y180H, A205T, R210P and R246C structure of CDK4-Cyclin D1 protein complex were found to be~22.216 nm^2^, ~26.786 nm^2^, ~32.264 nm^2^, ~36.569 nm^2^, ~27.987 nm^2^, and ~26.018 nm^2^ respectively (S5 Fig). All the mutant complexes showed high values suggesting an overall escalation in the flexibility than the native complex during the collective motion of the protein. The mutant protein complexes covered the larger region of conformational space than the native complex. From these projections, it was observed that clusters of mutants were well defined and was less stable compared to the native protein complex.

### Molecular dynamics simulation studies of native and mutant CDK4 structures in complex with flavopiridol and virtually screened compounds

To verify whether the results obtained by the molecular docking analysis were robust or fortuitous, we performed molecular dynamics simulations with native and mutant (R24C, Y180H, A205T, R210P, and R246C) protein-ligand complexes CDK4-flavopiridol, R24C-5_7_DIHYDROXY_ 2_ (3_4_5_TRIHYDROXYPHENYL) _4H_CHROMEN_ 4_ONE, Y180H-Diosmin, A205T-Rutin, R210P-Rutin, and R246C-5_7_DIHYDROXY_2_ (3_4_5_TRIHYDROXYPHENYL) _4H_CHROMEN_ 4_ONE.The atomic RMSD of the backbone atoms of the protein-ligand complexes obtained from the trajectories and the initial structures was monitored during the simulation(S6 Fig). A sharp rise was observed in the first ~ 20000ps and the function remained stable for the rest of the simulation. The system reached a constant temperature. The total energy was fluctuated around an average energy with the system stabilising at a temperature of 310K and one atmospheric pressure. The RMSD of the molecular dynamics simulation was stable after ~2000ps at the equilibrium. The native and mutant protein-ligand trajectories attained stable RMSD values around ~30000ps, indicating a high binding affinity between the protein-ligand complexes, further enhancing the credibility of the docking results.

**Fig 5:**
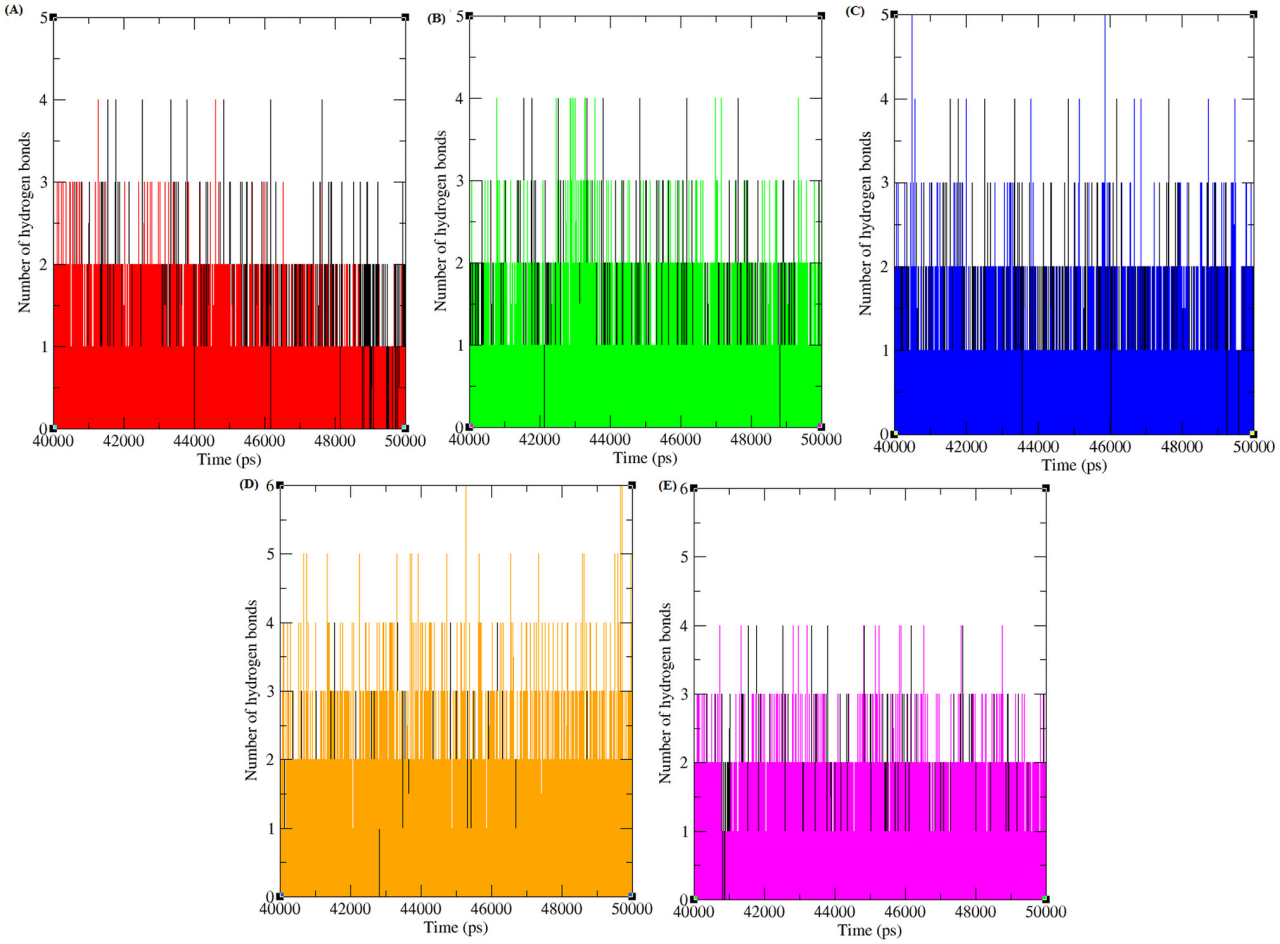
Total number of hydrogen bond formed between CDK4 and different inhibitors in native and mutant state. Black, Red, Green, Blue, Orange and Pink lines indicate the hydrogen bonds formed between CDK4-flavopiridol, R24C-5_7_DIHYDROXY_ 2_ (3_4_5_TRI HYDRO XYPHENYL) _4H_CHROMEN_ 4_ONE, Y180H-Diosmin, A205T-Rutin, R210P-Rutin and R246C-5_7_DIHYDROXY_2_ (3_4_5_TRIHYDROXYPHENYL) _4H_CHROMEN_ 4_ONE respectively.

Furthermore, to provide insight into the binding affinity of the protein and ligand, hydrogen bond contributions were analysed in the well equilibrated simulation period of the last 10 ns. The total number of hydrogen bonds between CDK4-flavopiridol and mutant CDK4-virtually screened compounds were analysed (Fig 5). CDK4-flavopiridol had one to four hydrogen bonds in the last 10ns simulation period. The numbers of hydrogen bonds formed between R24C-5_7_DIHYDROXY_ 2_ (3_4_5_TRI HYDRO XYPHENYL) _4H_CHROMEN_ 4_ONE, Y180H-Diosmin, A205T-Rutin, R210P-Rutin and R246C-5_7_DIHYDROXY_2_ (3_4_5_TRIHYDROXYPHENYL) _4H_CHROMEN_ 4_ONEmutant protein-ligand complexes were 1 to 4, 1 to 4, 1 to 5, 1 to 6 and 1 to 4, respectively. The number of hydrogen bonds between the mutant proteins R24C, Y180H, and R246C and their respective inhibitors is similar to the CDK4-flavopiridol complex. The mutant proteins A205T and R210P had a similar number of hydrogen bonds with the inhibitor rutin during the simulation period. These results provide evidence that these selected compounds have high capacity to function as strong inhibitors for various CDK4 protein mutants.

**Fig 6:**
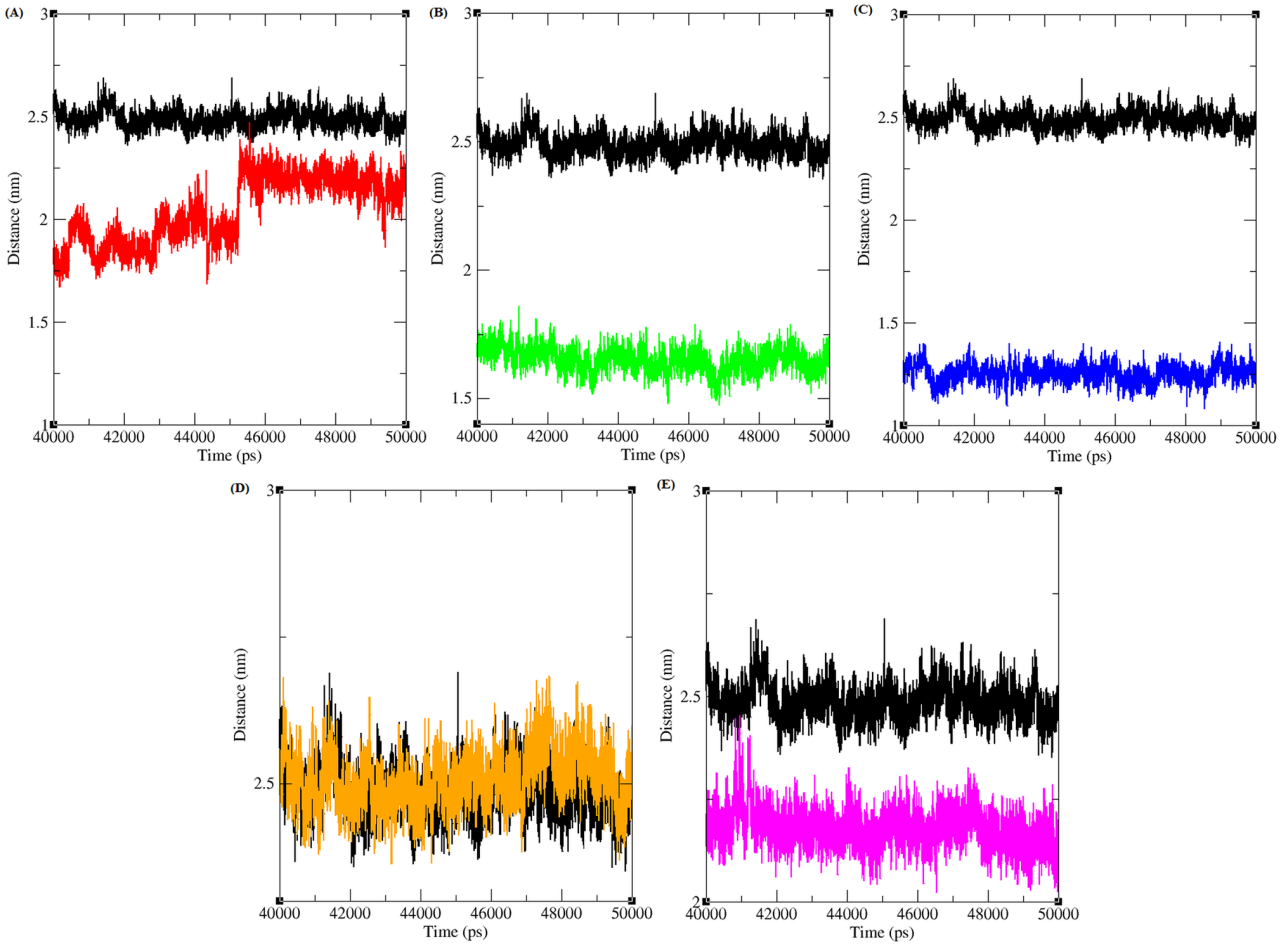
Minimum distance between CDK4 and different inhibitors in native and mutant state. Black, Red, Green, Blue, Orange and Pink lines indicate the hydrogen bonds formed between CDK4-flavopiridol, R24C-5_7_DIHYDROXY_ 2_ (3_4_5_TRI HYDRO XYPHENYL) _4H_CHROMEN_ 4_ONE, Y180H-Diosmin, A205T-Rutin, R210P-Rutin and R246C-5_7_DIHYDROXY_2_ (3_4_5_TRIHYDROXYPHENYL) _4H_CHROMEN_ 4_ONE respectively.

The minimum distance between CDK4-flavopiridol and mutant CDK4-virtually screened compounds was analysed (Fig 6). The minimum distance between CDK4-flavopiridol was ~2.5 nm in the last 10ns simulation period. The minimum distance between R24C-5_7_DIHYDROXY_ 2_ (3_4_5_TRI HYDRO XYPHENYL) _4H_CHROMEN_ 4_ONE, Y180H-Diosmin, A205T-Rutin, R210P-Rutin, and R246C-5_7_DIHYDROXY_2_ (3_4_5_ TRIHYDROXYPHENYL) _4H_CHROMEN_ 4_ONE mutant protein-ligand complexes was observed as ~1.55 to ~2.25, ~1.55, ~1.25,~2.5 and ~2.25, respectively. The distance between the mutant proteins and their respective inhibitor is less than for the native CDK4-flavopiridol complex, indicating that the screened inhibitor has a good binding affinity for the mutant proteins.

## Discussion

The endpoint of deleterious nsSNP identification should be protein structural and functional analysis. Proper understanding of protein conformational changes elucidates the mechanisms underlying disease phenotypes and aids in the identification of suitable drugs for structurally modified proteins. Deleterious nsSNPs cause protein structural and functional changes and are associated with diverse responses in drug efficacy in the human population. Therefore, these nsSNPs could occur in drug-binding target proteins and influence treatment outcomes. Rapid growth in the identification of SNPs makes it more difficult for experimental biologists to evaluate the biological significance of each SNP. However, computational, theoretical approaches can be used to identify and investigate the effects of deleterious polymorphisms that affect protein structure and function before undertaking further validation by experimental methods. Computational prediction methods such as SIFT, PolyPhen2 and I-Mutant 3.0 can screen for deleterious nsSNPs potentially involved in disease conditions. Drug targets for various diseases that are influenced by nsSNPs can be investigated using *in silico* methods, which has significant implications for specific target identification. Each computational pathogenicity prediction method follows a different strategy to estimate the effects of nsSNPs; hence, the prediction results are sometimes dissimilar. However, the positive predictions that overlap in the prediction methods should provide the greatest reliability to behave similarly. The variation in the identification of deleterious nsSNPs might be because of the differences in the adopted methodologies or the training datasets.

An in-depth knowledge of protein function could reveal the molecular mechanisms of the process that causes the disease condition. Deleterious nsSNPs in protein binding hot spots or the core regions of proteins can evidently disrupt protein interactions. The mapping of nsSNPs onto three-dimensional protein structures and analysing these changes at the structural level will help to find the exact point where they alter interactions with proteins. Three-dimensional models of mutant proteins are constructed by substituting the deleterious nsSNPs in the appropriate three-dimensional protein structures. Three-dimensional models are the simplest way to detect the types of harmful effects that deleterious nsSNPs can have on protein-protein and protein-ligand interactions. To obtain in-depth knowledge of protein structure and understand the adverse changes that these deleterious nsSNPs cause in CDK4 proteins, we extended our study by analysing the native and proposed mutant (protein-protein and protein-drug) structures at the atomic level by using a molecular dynamics simulation approach. Basic parameters such as RMSD, RMSF, hydrogen bond numbers, minimum distances, and SASA were analysed from the simulation trajectory values. Molecular stability and flexibility changes were observed from RMSD and RMSF. Stability is the fundamental property enhancing biomolecular function, activity, and regulation. Structural mutations affected buried residues in the protein core, causing changes in amino acid size, amino acid charge and hydrogen bond numbers [47]. Hydrogen bonds are the most important factor that creates a stable contact between a protein and its binding partner. Furthermore, number of studies have reported that protein interaction sites often have electrostatic complementarity with the charge distribution of the interacting partner [48–51]. Taken together, deleterious nsSNPs may affect the electrostatic charge distribution and alter the binding surfaces, causing the formation of more or fewer hydrogen bonds. Changes in protein stability, flexibility, hydrogen bonding, minimum distance between molecules and SASA have been shown to cause the loss of thermodynamic stability, as well as aberrant folding and aggregation of the protein [47]. Studies have indicated that many disease-related mutations lie in solvent-accessible sites, suggesting that the analysis of these mutations might also shed light on the mechanisms underlying disease conditions [52–57].

We performed a systematic computational analysis of the*CDK4* gene to identify potentially deleterious nsSNPs and their structural and functional significance, through molecular docking and molecular dynamics approaches. To predict the significant effect of nsSNPs in the*CDK4* gene, we applied three widely used computational methods, SIFT, PloyPhen2 and I-Mutant3, and each tool predicted 11, 9 and 15 nsSNPs as deleterious, respectively. Comparing the predictions made by all three methods, five amino acid variants (R24C, Y180H, A205T, R210P and R246C) of the CDK4protein were identified as highly deleterious, and all five variants were investigated structurally and functionally.For the structural analysis, we modelled the mutant structure with Spdbv software, after which energy minimization was performed for the native and mutant CDK4-cyclin D1 complexes using a steepest descent force field to check and repair the geometry of the modelled complex. Next, docking analysis was performed between the native and mutant structures of CDK4 with flavopiridol. Several factors enhancing the protein-ligand interactions were analysed. Binding energies between the mutant protein and ligand revealed a lower binding capability of the mutant structures to the drug flavopiridol. Notably, flavopiridol interacting residues differed in the mutant proteins compared with native CDK4 protein. Although the mutant structures R24C, A205T, R210P, and R246C obtained a similar number of hydrogen bonds with flavopiridol, the ligand failed to bind to the ATP-binding site of the mutant proteins. These results indicate that the structural changes in the CDK4protein occurred because of the substitution of deleterious amino acids. Another functionally significant property of the protein was explored through the docking of flavopiridol with CDK4, demonstrating the effect of fluctuation in protein-ligand complex formation. A change in the pattern of flexibility of the functional residues might alter the functional region and activity of mutant proteins. A decrease in overall flexibility and an increase in rigidity due to mutations may affect the binding properties of proteins. To identify a suitable inhibitor for mutant proteins, structure-based virtual screening and docking analysis were performed. A total of 19 compounds that were structurally similar to flavopiridol were retrieved from the DrugBank database. All of the compounds were individually docked with theR24C, Y180H, A205T, R210P and R246C mutant structures of the CDK4 protein. The inhibitor 5_7_DIHYDROXY_ 2_ (3_4_5_TRI HYDRO XYPHENYL) _4H_CHROMEN_ 4_ONE displayed good binding affinity and inhibition at the ATP-binding site of R24C and R246C.Diosmin displayed good binding affinity and interaction with the ATP-binding residue of Y180H. Rutin displayed good binding affinity and interaction with the A205T and R210P mutant proteins.

Furthermore, we performed 50ns MD simulation analysis on the native and mutant (R24C, Y180H, A205T, R210P, and R246C) protein-protein and protein-ligand complexes. MD simulation analysis provides in-depth knowledge regarding the protein-protein and protein-ligand interactions at the atomic level. Five fundamental parameters (RMSD, RMSF, hydrogen bond numbers, minimum distances and SASA) were examined during the last 10 ns of the CDK4-Cyclin D1 simulation trajectories for the native and mutant complexes. Molecular stability and flexibility changes were observed through RMSD and RMSF analysis. Stability is the primary property that enhances biomolecular function, activity, and regulation. The results obtained from the CDK4-Cyclin D1complex stability analysis concluded that all five mutant complexes had different RMSD values from the native complex. A larger deviation increases the stability of molecules and vice versa. A higher stability increases the rigidity of the protein, and a lower stability increases the flexibility of the protein. Henceforth, from the stability analyses of native and mutant CDK4-CyclinD1protein complexes, it was observed that the mutant complexes had different stabilities because of the substitution of deleterious amino acids. From the RMSF analyses, we observed changes in flexibility for all five mutant complexes. Based on the results of the RMSD and RMSF analyses, we confirmed that the substitution of amino acids adversely affected the stability and flexibility of CDK4-CyclinD1 mutant complexes. In addition to the different electrostatic forces, the hydrogen bonds across the protein-protein interacting interface act as the main contributor in maintaining the stability of the protein. Furthermore, the presence of deleterious polymorphisms might change the hydrogen bond formation between the molecules. Consequently, in all five mutant complexes less number of hydrogen bonds was observed between the CDK4-Cyclin D1 proteins. A decrease in the number of hydrogen bonds revealed that the binding stability of the CDK4-Cyclin D1 mutant complexes might be affected. The minimum distance between CDK4 and CyclinD1 in the protein-protein complexes was analysed for both the native and mutant CDK4-Cyclin D1 complexes. The distance between the CDK4 and Cyclin-D1 proteins increased in all five mutant complexes compared with the native complex. An increase in the distance might reduce the binding affinity between the CDK4-CyclinD1 mutant complexes. Furthermore, in the SASA analysis, with respect to the native protein, different areas of solvent accessible surface were observed in all five mutant CDK4 proteins. The differences in the accessible areas in mutant proteins might alter the probability of an interaction between CDK4 in Cyclin D1 protein complexes. Subsequent SASA analysis revealed that the presence of deleterious polymorphisms in the CDK4 protein might change the hydrophilic and hydrophobic areas of the mutant CDK4 proteins.

For CDK4-flavopiridol, R24C-5_7_DIHYDROXY_ 2_ (3_4_5_TRI HYDRO XYPHENYL) _4H_CHROMEN_ 4_ONE, Y180H-Diosmin, A205T-Rutin, R210P-Rutin and R246C-5_7_DIHYDROXY_2_ (3_4_ 5_ TRIHYDROXYPHENYL) _4H_CHROMEN_ 4_ONE, docked protein-ligand complexes were subjected to MD simulation for 50 ns to analyse RMSD, H-bonds, and minimum distances. In the molecular stability change analysis, all five protein-ligand complexes averaged lower RMSD values than those of the native protein-ligand complex. Hydrogen bond interactions between the protein and ligand served as the main contributor in maintaining the stable contact between the molecules. The substitution of deleterious nsSNPs might change the electrostatic charge distribution in proteins and affect the normal protein-ligand interactions. However, the virtually selected inhibitors exhibited good binding affinity for the mutant proteins, having the potential to maintain a stable number of hydrogen bonds during the simulation period. In all five mutant complexes (R24C, Y180H, A205T, R210P, and R246C), a similar number of H-bonds was observed between the proteins and their respective virtually selected ligand molecules. The distances between the mutant proteins and their respective virtually screened potential ligands were consistently lower than that measured for the native CDK4-flavopiridol complex. A reduction in this distance may increase the affinity of the mutant CDK4 proteins for their respective virtually screened compounds.

## Conclusion

Alterations in protein structure are mainly caused by deleterious nsSNPs in the nucleotide sequence. The occurrence of deleterious nsSNPs in the genome is relatively rare but has a significant effect on protein structure and function. A number of computational methods have been designed to predict the functional effects of nsSNPs whether a particular nsSNP is a driver of carcinogenesis. In this study, by following an integrated computational approach, we identified and investigated the effects of harmful polymorphisms at both the structural and functional level for the *CDK4* gene. Furthermore, this study highlights successful drug repositioning in mutant CDK4 proteins in the event of conformational changes attributable to deleterious nsSNPs. This information on drug repositioning can accelerate the development of cancer drugs, and this study of new cancer drug targets can potentially be used for future target identification. The identification of new drug targets and new uses for existing drugs hold promise for the future use of computational methods in cancer research. Overall, the methodology adopted in this study is extremely useful in the drug discovery process and will improve drug efficacy and safety profiles in cancer treatment.

## Supporting Information

S1 Fig Ligplot analysis of CDK4-Flavopiridol complex in both native and mutant state. (A) Native complex is showing high number of residues interacting with the drug flavopiridol. (B) Ligplot showing the interaction between mutant model R24C and flavopiridol. (C) Ligplot showing the interaction between mutant type Y180H and flavopiridol. (D) Ligplot showing the interaction between mutant type A205T and flavopiridol. (E) Ligplot showing the interaction between mutant model R210P and flavopiridol. (F) Ligplot showing the interaction between mutant type R246C and flavopiridol.
(TIF)

S2 Fig Backbone Root Mean Square Deviation (RMSD) of CDK4 protein in complex with cyclin D1. The ordinate is RMSD (nm), and the abscissa is the time (ps). Black, Red, Green, Blue, Orange, and Pink lines indicate native, R24C, Y180H, A205T, R210P, and R246C protein complexes respectively.
(TIF)

S3 Fig C-alpha Root Mean Square Fluctuation (RMSF) of CDK4 protein in complex with cyclin D1. The ordinate is RMSF (nm) and the abscissa amino acid residues. Black, Red, Green, Blue, Orange, and Pink lines indicate native, R24C, Y180H, A205T, R210P and R246C protein complexes respectively.
(TIF)

S4 Fig Solvent accessible surface area (SASA) analysis of native and mutant CDK4-cyclin D1 complexes. Black, Red, Green, Blue, Orange and pink lines indicate native, R24C, Y180H, A205T, R210P, and R246C CDK4-Cyclin D1 protein complexes.
(TIF)

S5 Fig Projection of the motion of proteins in phase space along the first two principal eigenvectors. Black, Red, Green, Blue, Orange and pink lines indicate native, R24C, Y180H, A205T, R210P, and R246C CDK4-Cyclin D1 protein complexes.
(TIF)

S6 Fig Backbone Root Mean Square Deviation (RMSD) of CDK4 protein in complex with selective inhibitors. The ordinate is RMSD (nm), and the abscissa is the time (ps). Black, Red, Green, Blue, Orange and Pink lines indicate native, R24C, Y180H, A205T, R210P, and R246C protein-ligand complexes respectively.
(TIF)
