# Supplementary figures and images for: Analysing the Effect of Mutation on Protein Function and Discovering Potential Inhibitors of CDK4: Molecular Modelling and Dynamics Studies
Source: PLoS One. 2015 Aug 7;10(8):e0133969. doi: 10.1371/journal.pone.0133969 (PMC4529227; doi:10.1371/journal.pone.0133969)

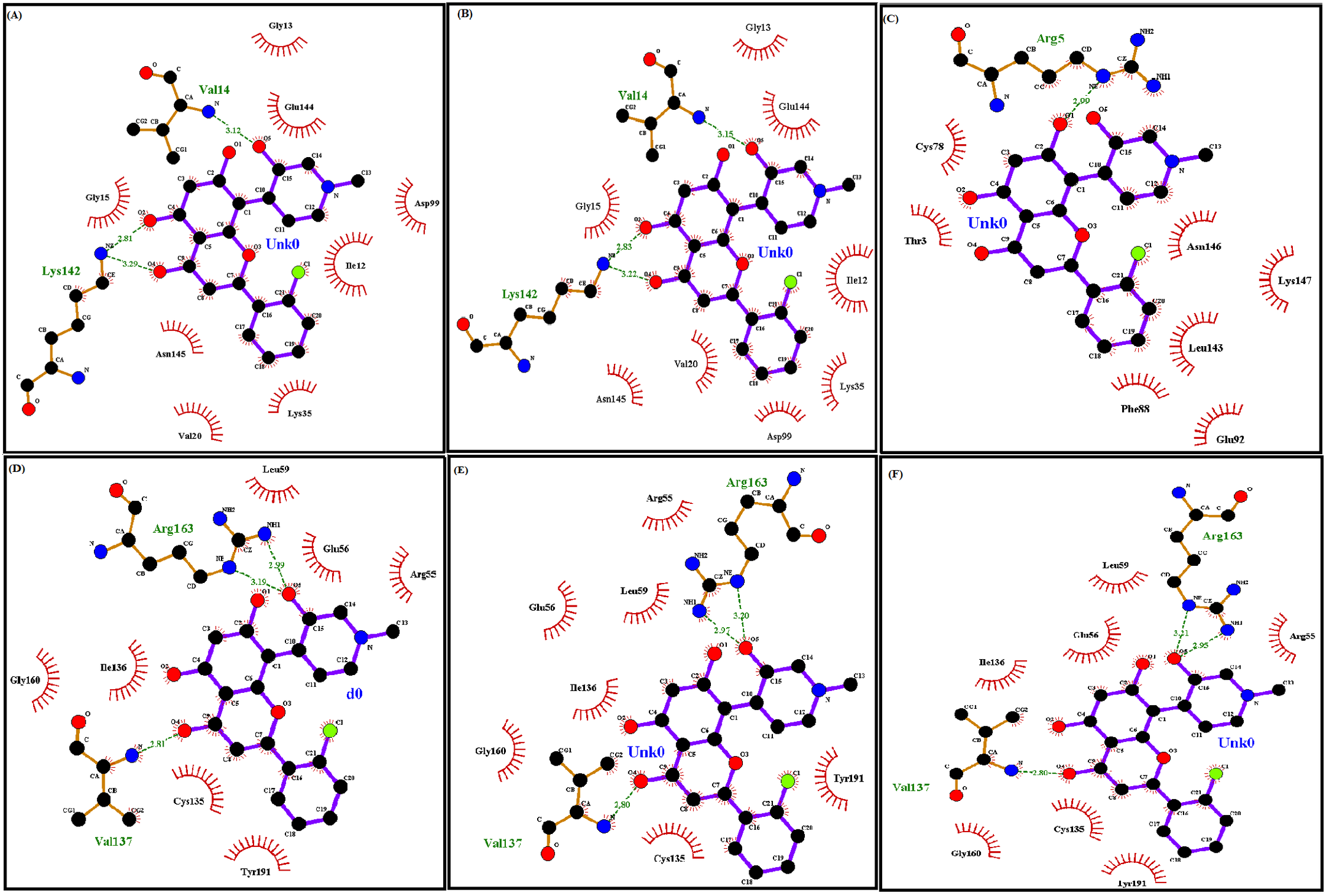

Supplement: S1 Fig — (A) Native complex is showing high number of residues interacting with the drug flavopiridol. (B) Ligplot showing the interaction between mutant model R24C and flavopiridol. (C) Ligplot showing the interaction between mutant type Y180H and flavopiridol. (D) Ligplot showing the interaction between mutant type A205T and flavopiridol. (E) Ligplot showing the interaction between mutant model R210P and flavopiridol. (F) Ligplot showing the interaction between mutant type R246C and flavopiridol. (TIF) [file pone.0133969.s001.tif]

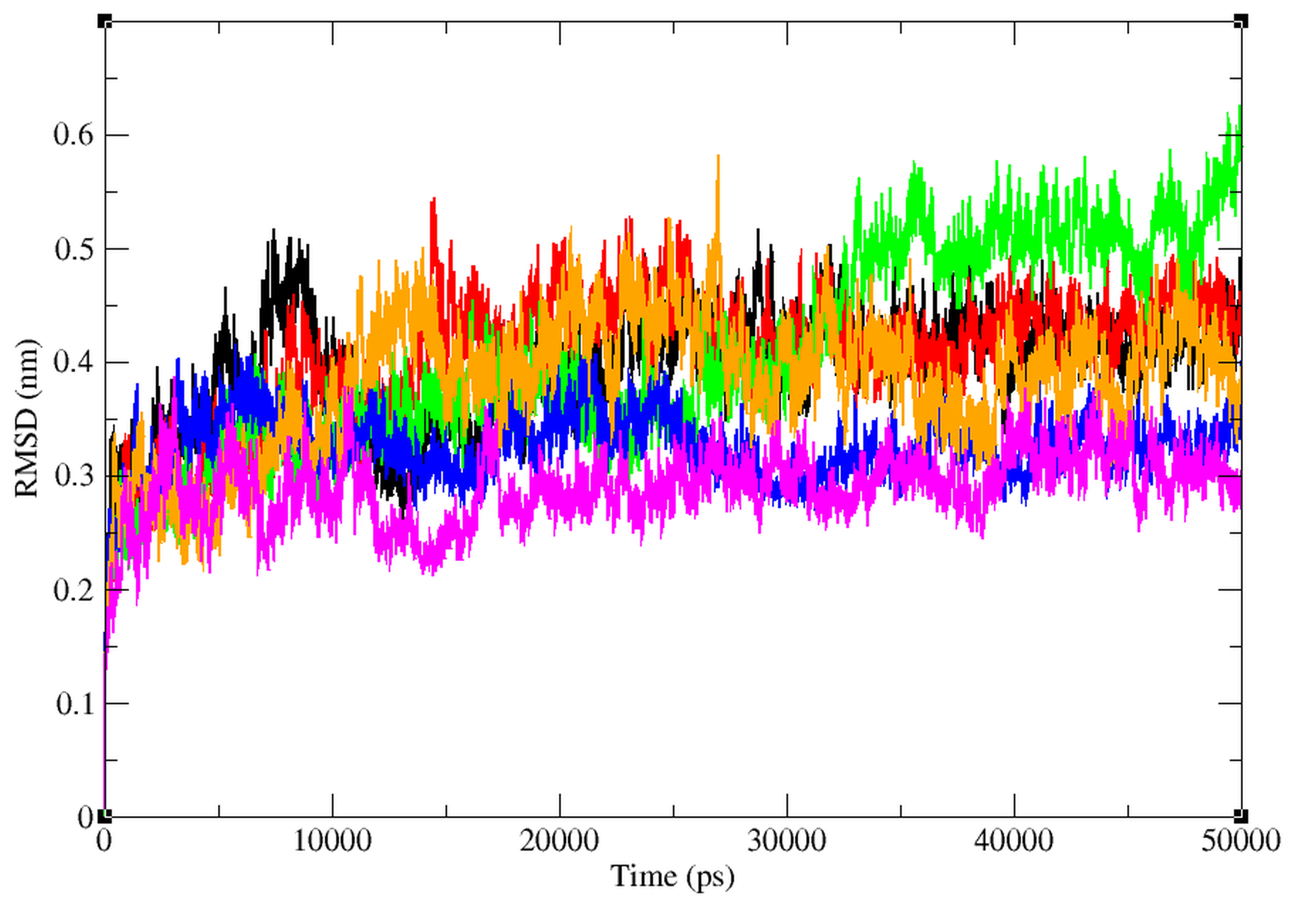

Supplement: S2 Fig — The ordinate is RMSD (nm), and the abscissa is the time (ps). Black, Red, Green, Blue, Orange, and Pink lines indicate native, R24C, Y180H, A205T, R210P, and R246C protein complexes respectively. (TIF) [file pone.0133969.s002.tif]

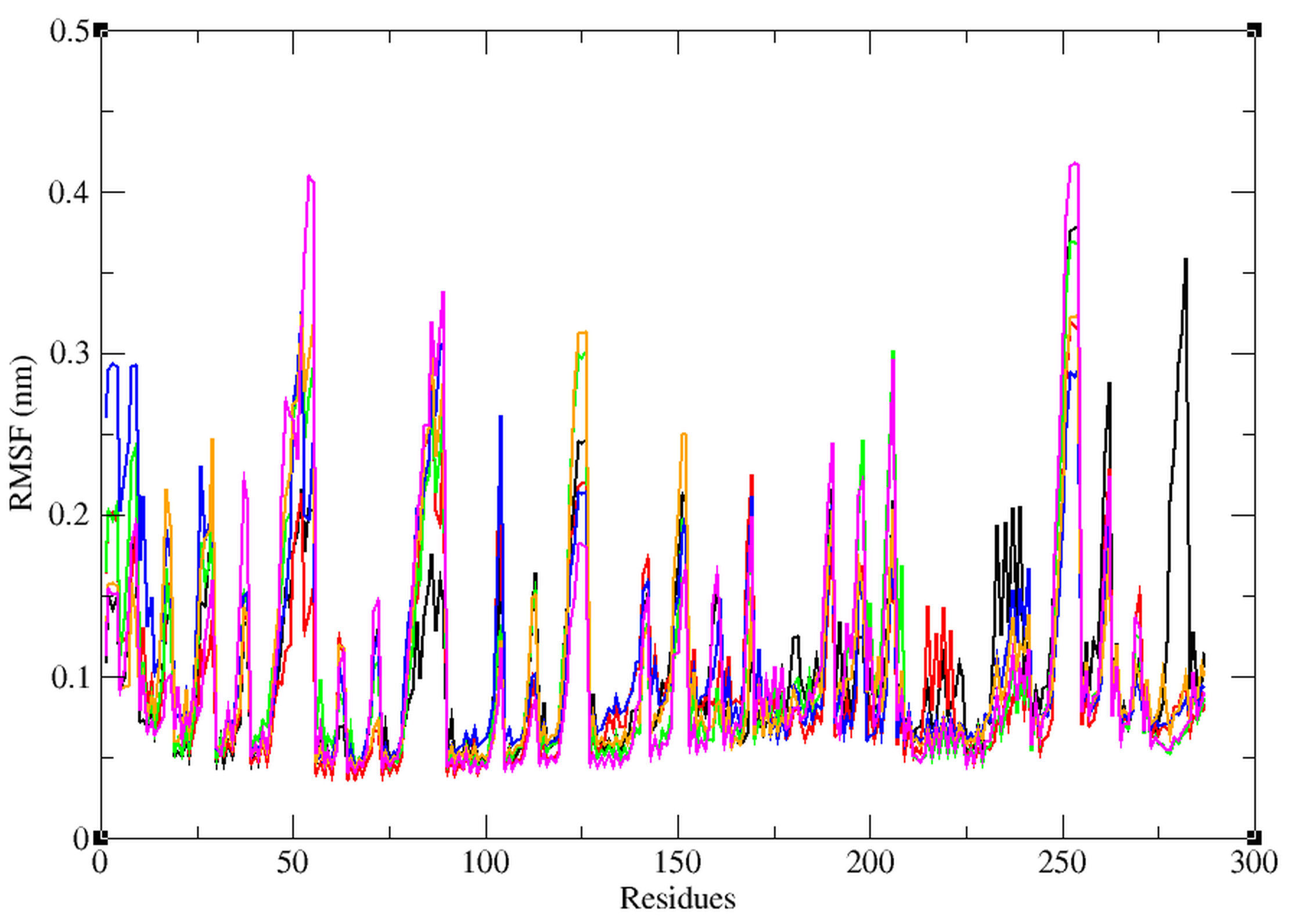

Supplement: S3 Fig — The ordinate is RMSF (nm) and the abscissa amino acid residues. Black, Red, Green, Blue, Orange, and Pink lines indicate native, R24C, Y180H, A205T, R210P and R246C protein complexes respectively. (TIF) [file pone.0133969.s003.tif]

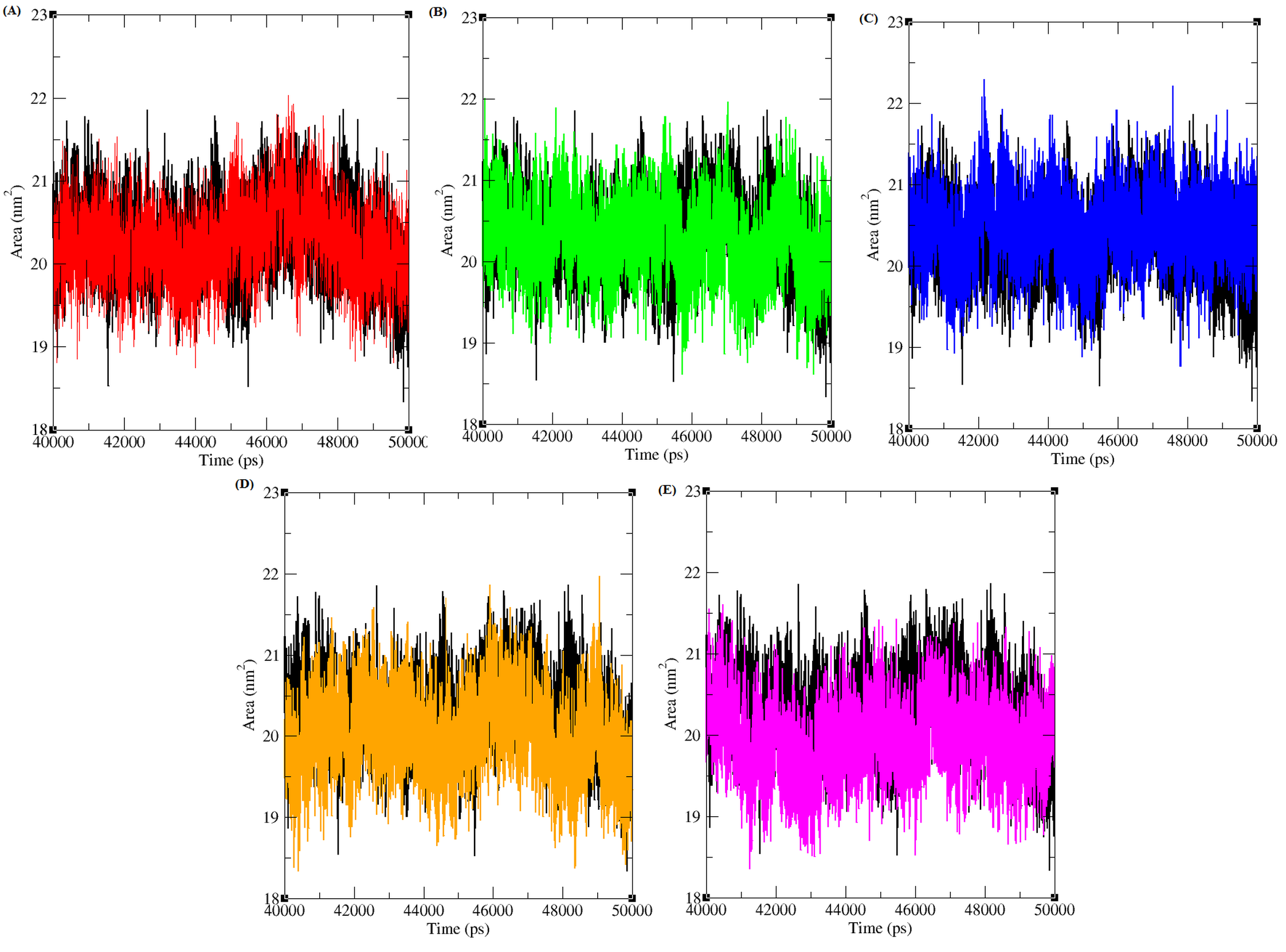

Supplement: S4 Fig — Black, Red, Green, Blue, Orange and pink lines indicate native, R24C, Y180H, A205T, R210P, and R246C CDK4-Cyclin D1 protein complexes. (TIF) [file pone.0133969.s004.tif]

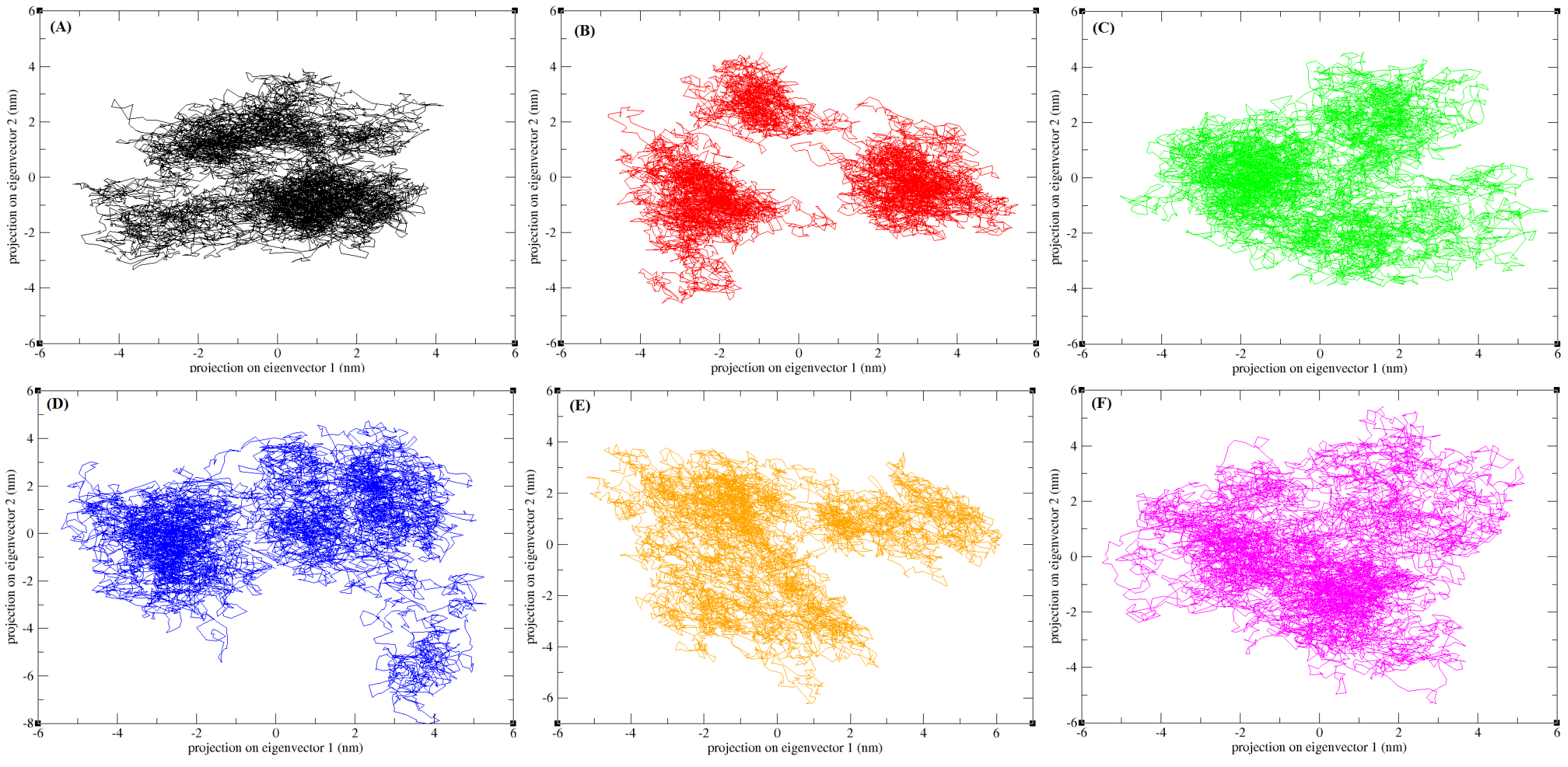

Supplement: S5 Fig — Black, Red, Green, Blue, Orange and pink lines indicate native, R24C, Y180H, A205T, R210P, and R246C CDK4-Cyclin D1 protein complexes. (TIF) [file pone.0133969.s005.tif]

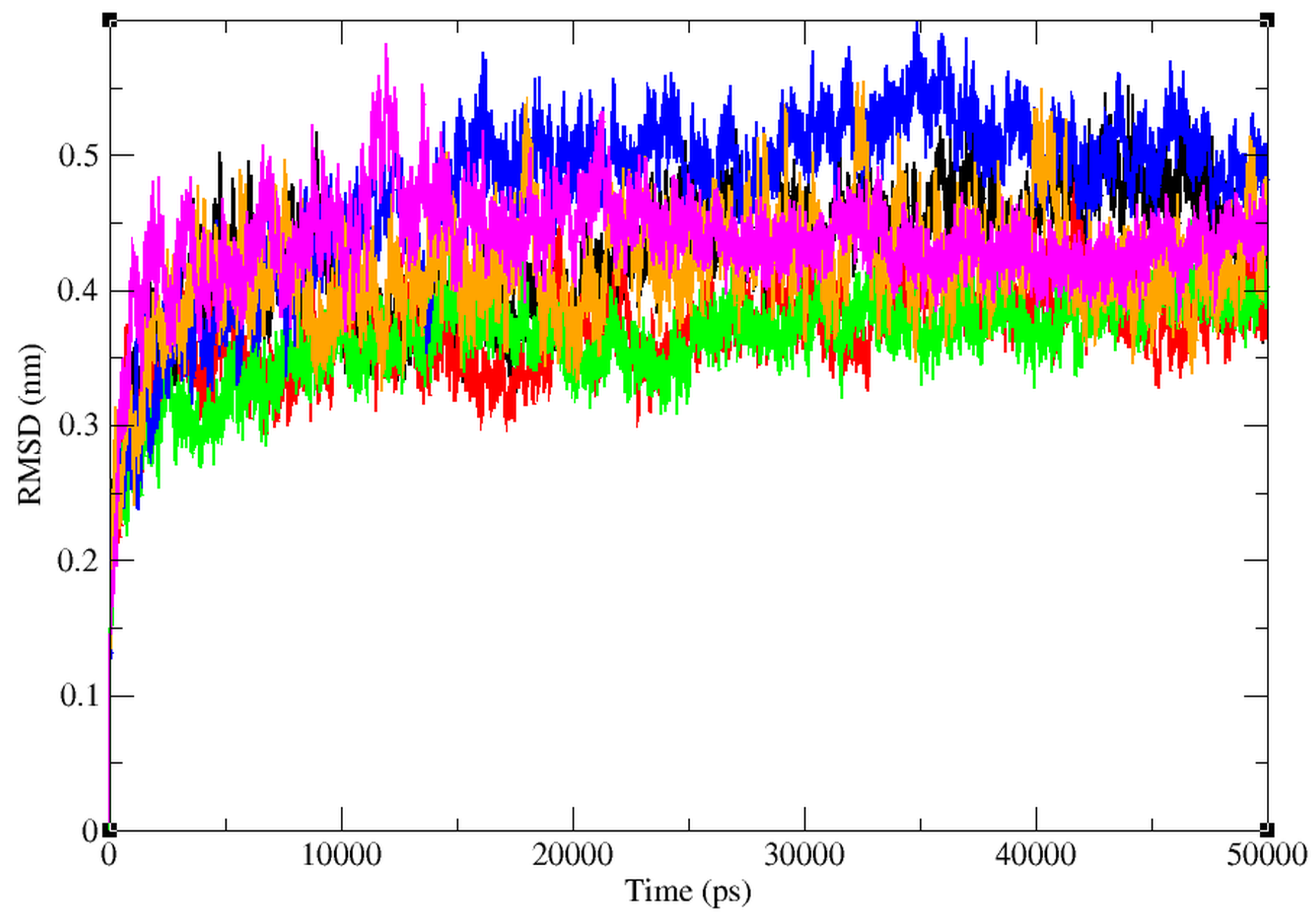

Supplement: S6 Fig — The ordinate is RMSD (nm), and the abscissa is the time (ps). Black, Red, Green, Blue, Orange and Pink lines indicate native, R24C, Y180H, A205T, R210P, and R246C protein-ligand complexes respectively. (TIF) [file pone.0133969.s006.tif]
